# Anisotropic honeycomb stack metamaterials of graphene for ultrawideband terahertz absorption

**DOI:** 10.1515/nanoph-2023-0500

**Published:** 2023-11-06

**Authors:** Xueying Liu, Yinong Xie, Jinlin Qiu, Wei Chen, Yineng Liu, Jinfeng Zhu

**Affiliations:** Institute of Electromagnetics and Acoustics and Key Laboratory of Electromagnetic Wave Science and Detection Technology, Xiamen University, Xiamen 361005, China; Key Laboratory of Grain Information Processing and Control, College of Information Science and Engineering, Henan University of Technology, Zhengzhou 450001, China; Shenzhen Research Institute of Xiamen University, Shenzhen 518057, China

**Keywords:** terahertz, metamaterial, anisotropic graphene aerogel, broadband absorption

## Abstract

Graphene aerogels have implied great potential for electromagnetic wave absorption. However, the investigation of their design for broadband absorption in the terahertz (THz) range remains insufficient. Here, we propose an anisotropic honeycomb stack metamaterial (AHSM) based on graphene to achieve ultrawideband THz absorption. The absorption mechanism is elucidated using the effective medium method, offering deeper physics insights. At low THz frequencies, the impedance matching from the air to the AHSM can be improved by reducing the chemical potential of graphene for high absorption. There is a suppression of absorption at the intermediate frequencies due to constructive interference, which can be avoided by shortening the sizes of honeycomb edges. With the aim to elevate absorption at high frequencies, one can increase the stack layer number to enhance multiple reflections and destructive interference within the metastructure. Based on the above principles, we design an AHSM that achieves a broadband absorbance of over 90 % from 1 THz to 10 THz. This absorption can tolerate a wide range of incident angles for both TE and TM wave excitations. Our research will provide a theoretical guide to future experimental exploration of graphene aerogels for THz metamaterial absorber applications.

## 1 Introduction

Efficient terahertz (THz) wave-absorbing materials are quite in demand for the fast-growing THz applications, including imaging [1], spectroscopy [2], high-speed communication [3], and detection [4]. A variety of THz wave-absorbing materials are pursued for future electromagnetic environments, which typically should have high THz absorption intensity, wideband operating bandwidth, and angle insensitivity [5–10]. Graphene, a unique and attractive class of carbon-based material with remarkable mechanical, electronic, and optoelectronic properties, has shown promising potential for electrical and optical devices [11–15]. Particularly, graphene-based metamaterial THz absorbers have been extensively investigated driven by a steady pace of nanofabrication development and increasing attention [16–19]. To increase the absorption performance and bandwidth of graphene-based MMs, researchers have incorporated multiple resonant arrays within a one-unit cell, resulting in the emergence of dual-band, triple-band, and multi-band absorbers [20–25]. However, these configurations face shortcomings such as limited bandwidths, complicated designs, and expensive device nanofabrication.

Graphene aerogels (GAs) have emerged as one of the most promising candidates for lightweight high-performance electromagnetic wave absorption materials, which keep the excellent properties of single-layer graphene sheets [26–30]. The porous structures in GAs provide rich electromagnetic interactions, including giant cross-linked electric loss networks, multiple internal reflections, and scattering; these properties enhance the attenuation of incident electromagnetic waves for achieving perfect absorption [31, 32]. Particularly, GAs with anisotropic highly arranged microstructures have attracted much more attention [33, 34]. By freezing of graphene hydrogels followed by freeze-drying, researchers have prepared anisotropic graphene aerogels (AGAs) and studied their electromagnetic wave shielding properties at microwave frequencies [35, 36]. The AGAs with honeycomb-like and lamellar microstructures have demonstrated the extraordinary features of electromagnetic absorption in the perpendicular direction [37–42]. Designing macrocellular architecture and understanding the structure-performance relationships are of scientific significance for realizing satisfactory THz absorbers based on graphene. So far, there is a lack of work on investigating the absorption properties of AGAs over the THz range, which hinders the fast development of high-performance devices.

In this work, we propose a kind of anisotropic honeycomb stack metamaterial (AHSM) based on graphene to achieve THz ultrawideband absorption, drawing inspiration from the properties of AGAs. The physics of wave absorption under different THz frequencies is elucidated by field distributions, impedance matching theory, and effective medium theory (EMT). At low frequencies, high absorption can be achieved by reducing the chemical potential of graphene, due to the optimal impedance matching. To decrease the interference of absorption dips at intermediate frequencies, one can reduce honeycomb edge size to avoid the constructive interference. The stack layer number can be increased to enhance the multiple reflection and destructive interference for elevated absorption at high frequencies. Based on the above theory, we achieve ultrabroadband absorption of more than 90 % from 1 THz to 10 THz with good robustness on incident angle and polarization. The proposed anisotropic absorbers are promising for applications on various THz sensing, communication, and imaging systems.

## 2 Design and method

**Figure 1:**
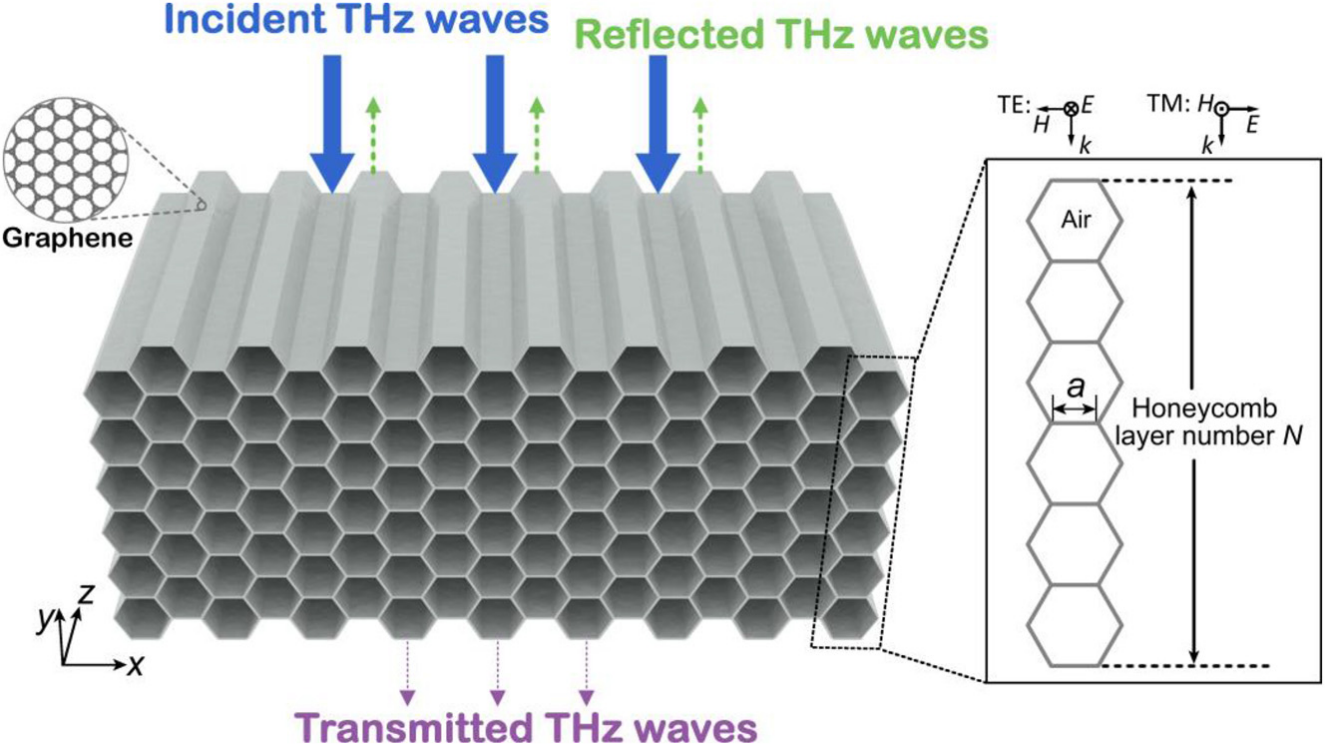
Schematic drawing for the AHSM. The symbols *a* and *N* denote the honeycomb edge size and honeycomb layer number, respectively.

The AHSM based on graphene consists of a regular hexagonal array, as depicted in Figure 1. The unit cells repeat periodically in the *x* direction, with the structure extending infinitely along the *z* direction. Each hexagon’s edge represents the graphene material, while the interior of each hexagon is filled with air. The size of the honeycomb cell is determined by the length of the honeycomb edge *a*, and the thickness of the absorber structure is determined by the number of multi-stacked layers *N*. By tuning the freeze casting temperature, the honeycomb size *a* can be customized [38].

**Figure 2:**
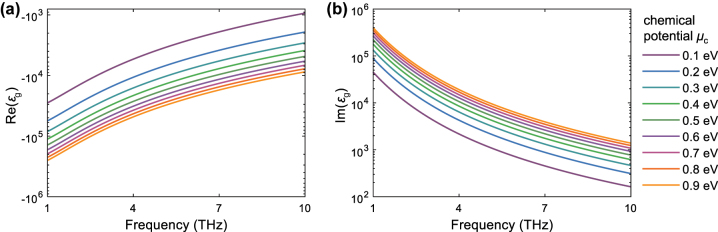
Graphene permittivity as a function of frequency and chemical potential *μ*_*c*_ for (a) real part and (b) imaginary part.

We perform optical simulations based on the finite element method via the commercial software COMSOL Multiphysics. The incident wave is introduced from the top of metamaterial, as shown in Figure 1. Floquet boundary conditions are used for the periodic unit cells. In the theoretical model, the monolayer graphene is adopted. It can be used as the surface conductivity boundary since it is a two-dimensional film with a thickness of 0.34 nm, which is much smaller than the smallest THz wavelength. The surface electrical conductivity *σ*_*g*_ (*ω*, *μ*_*c*_, Γ, *T*) of graphene is contributed by intra-band and inter-band electron transitions (*σ*_*g*_ = *σ*_intra_ + *σ*_inter_). In the THz range, *σ*_*g*_ can be described by the Kubo equation as below [43–46],

(1) $$\begin{array}{ll} \sigma_{\text{g}}\left(\omega ,\mu_{c},\tau ,T\right) & =i\frac{e^{2}k_{B}T}{\pi \hbar^{2}{\left(\omega +i\tau^{-1}\right)}^{2}}\\ & \;\times \left[\frac{\mu_{c}}{k_{B}T}+2ln\left[e^{-\frac{\mu_{c}}{k_{B}T}}+1\right]+i\frac{e^{2}}{4\pi \hbar }\ln\right.\\ & \;\times \left.\left[\frac{2\left|\mu_{c}\right|-\hbar \left(\omega +i\tau^{-1}\right)}{2\left|\mu_{c}\right|+\hbar \left(\omega +i\tau^{-1}\right)}\right]\right] \end{array}$$

where *ω* = *2πf* is the angular frequency, *μ*_*c*_ is the chemical potential of graphene, *ℏ* is the reduced Plank’s constant, *k*_B_ is the Boltzmann’s constant, and *e* is the electron charge. In the THz band, the relaxation time *τ* = 0.1 ps and the temperature *T* = 300 K [47]. The electrical conductivity *σ*_*g*_ and in-plane permittivity *ε*_*g*_ of graphene are related as follows [48, 49],

(2) $$\varepsilon_{g}=1+i\frac{\sigma_{g}}{\omega \Delta \varepsilon_{0}}$$

where *ε*_0_ is the vacuum permittivity and Δ = 0.34 nm represents the thickness of graphene. We plot the imaginary and real parts of the permittivity of graphene as a function of chemical potential and frequency, as shown in Figure 2. It shows that graphene’s permittivity gradually rises with its chemical potential.

## 3 Result and discussion

### 3.1 Effect of honeycomb edge and layer number on the absorption performance

**Figure 3:**
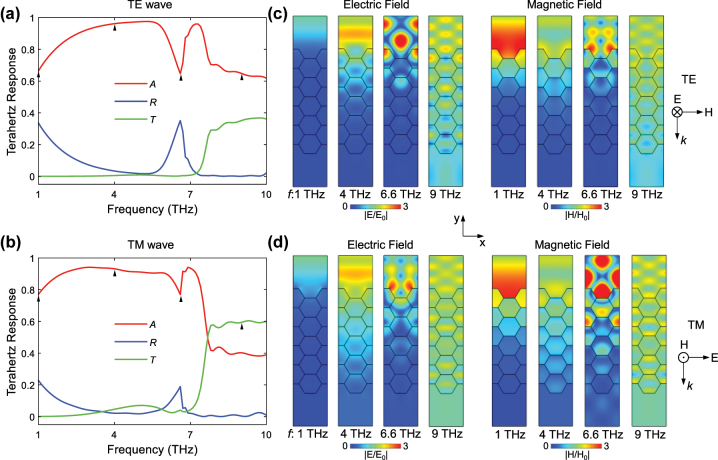
THz spectral response and the corresponding electromagnetic field distributions for (a), (c) TE and (b), (d) TM wave excitations, where *a* = 15 μm, *N* = 5, *μ*_*c*_ = 0.7 eV.

Initially, we have a rough overview of the THz spectral responses of the proposed metamaterial. We plot the spectra of absorbance (*A*), transmittance (*T*), and reflectance (*R*) of AHSM for *N* = 5 and *a* = 15 μm under TE and TM wave excitations in Figure 3(a) and (b), which indicates the similar spectral feature. In order to elucidate the THz physics, we analyze the electromagnetic field distributions of AHSM at four characteristic frequencies in Figure 3(c) and (d). At *f* = 1 THz, the electric and magnetic fields are mainly distributed above the metamaterial. A portion of THz wave is reflected at the surface of the AHSM, while another part is absorbed. The corresponding absorbance and reflectance are 66.2 % and 33.8 % for TE wave, respectively, and are 77 % and 23 % for TM wave, respectively. At *f* = 4 THz, the field is distributed in the upper half of metamaterial, and its intensity gradually decreases from top to bottom. Most of the THz wave penetrates into the metamaterial, and is mainly dissipated in its upper part; thus, the absorbance ratios of TE and TM waves are as high as 96.2 % and 93.2 %, respectively. At the absorption dip of *f* = 6.6 THz, the fields are enhanced and demonstrate specific patterns on top of the AHSM. This is because the interface between the AHSM and air forms a grating-like periodic structure, and leads to constructive interference between the outgoing and incoming waves. Such effect increases the reflection and lowers the absorption significantly. At *f* = 9 THz, there is a uniform electromagnetic field distribution in the AHSM, which indicates that the THz wave can transmit through the entire metamaterial. Under TE and TM waves, the reflectance ratios are almost zero, while the absorbance ratios are only 65 % and 41 %, respectively. The different spectral responses mainly result from the different effective resonance lengths in *x*- and *z*-directions. As the working frequency gets higher towards 10 THz, the THz wavelength becomes much shorter and closer to the AHSM aperture size, which results in the specific field pattern in and outside the metastructure. It indicates the destructive interference for zero reflectance.

**Figure 4:**
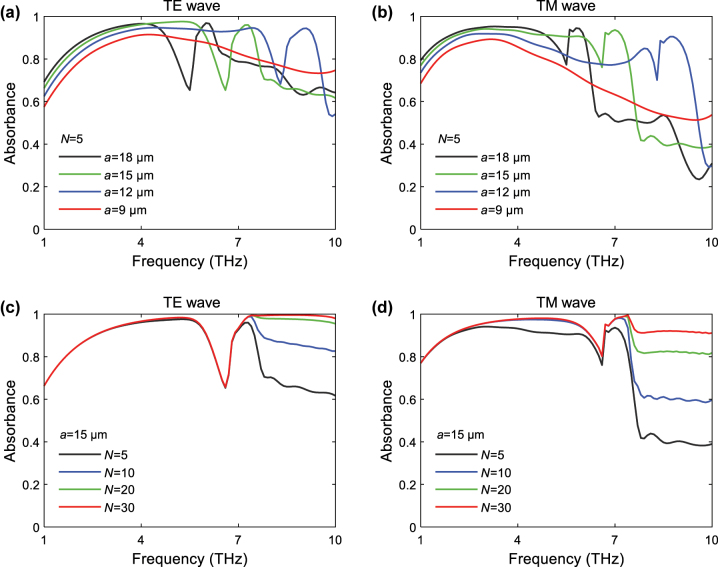
Absorbance of the AHSM with *N* = 5 and different *a* value under (a) TE and (b) TM wave excitations. Absorbance of the AHSM with *a* = 15 μm and different *N* values under (c) TE and (d) TM wave excitations, where *μ*_*c*_ = 0.7 eV.

We continue to investigate the effects of different *a* and *N* values on the THz absorption dips. As shown in Figure 4(a) and (b), the absorption dip emerges and shifts from high frequencies to low frequencies with the increase of *a*. The frequencies of the absorption dips are 8.3 THz, 6.6 THz, and 5.5 THz for *a* = 12 μm, 15 μm, and 18 μm, respectively. Notably, the wavelength corresponding to the dip frequency is about 3 times the size of the honeycomb edge, for instance, 36.14 μm versus 3 × 12 μm, 45.45 μm versus 3 × 15 μm, 54.55 μm versus 3 × 18 μm. This should be attributed to the interference by the hexagonal interfaces in the honeycomb topology with the specific honeycomb edge size. In contrast, for the AHSM with a fixed *a* value of 15 μm, the absorption dip around *f* = 6.6 THz barely changes with the increase of *N*, as shown in Figure 4(c) and (d). These results imply that the honeycomb edge has more dominant effects than the honeycomb layer number on tuning the absorption dips.

**Figure 5:**
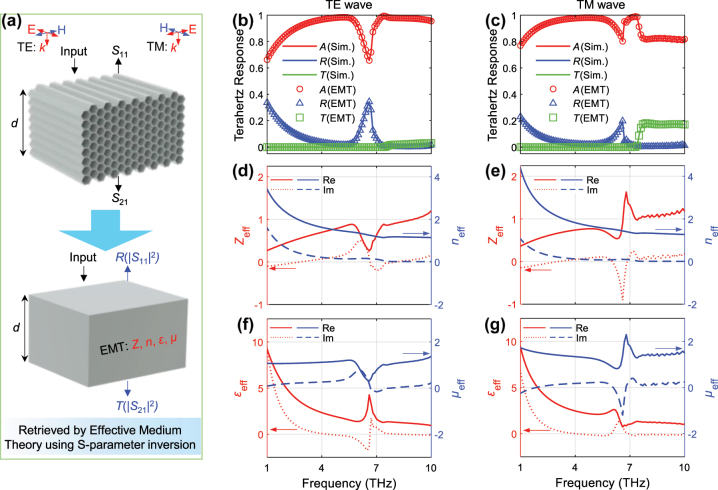
Effective optical properties of AHSM. (a) The schematic concept of retrieving the effective electromagnetic parameters of AHSM. Retrieved and simulated THz responses under (b) TE and (c) TM wave excitations. The effective complex optical parameter *Z*_eff_, *n*_eff_, *ε*_eff_, *μ*_eff_ of AHSM under (d) (f) TE and (e) (g) TM wave excitations, where *a* = 15 μm, *N* = 20 and *μ*_*c*_ = 0.7 eV.

In order to further reveal the absorption mechanism, we apply the effective medium method (EMT) by using *S*-parameter inversion [50–53]. Since the unit cell dimension of AHSM with *a* = 15 μm is smaller than the THz wavelength, the metamaterial can be treated as a homogenous slab with effective electromagnetic parameters, as summarized in Figure 5(a). The effective material parameters can be determined by the following equations,

(3) $$Z_{\text{eff}}=\sqrt{\frac{\mu_{\text{eff}}}{\varepsilon_{\text{eff}}}}=\sqrt{\frac{{\left(1+S_{11}\right)}^{2}-S_{21}^{2}}{{\left(1-S_{11}\right)}^{2}-S_{21}^{2}}}$$

(4) $$n_{\text{eff}}=\frac{1}{kd}\cos^{-1}\left[\frac{1}{2S_{21}}\left(1-S_{11}^{2}+S_{21}^{2}\right)\right]$$

(5) $$\varepsilon_{\text{eff}}\;=\;n_{\text{eff}}\text{/}Z_{\text{eff}}\text{, }\mu_{\text{eff}}\;=\;n_{\text{eff}}Z_{\text{eff}}$$

where *Z*_eff_, *n*_eff_, *ε*_eff_, *μ*_eff_ represent the effective impedance, refractive index, electric permittivity, and magnetic permeability, respectively. *d* and *k* represent the thickness of the metamaterial and the wavenumber of the free space. *S*_11_ and *S*_21_ are the reflectance and transmittance coefficients, which can be calculated from the commercial software COMSOL Multiphysics. The effective THz responses of the effective slab based on EMT are in good agreement with those from metamaterial simulation, as illustrated in Figure 5(b) and (c). For the frequency range (2.5–6 THz, 6.9–10 THz) where the absorbance exceeds 90 %, the corresponding real and the imaginary parts of *Z*_eff_ approach 1 and 0, respectively, as shown in Figure 5(d). This indicates that the impedance of AHSM is perfectly matched with that of the free space. Besides, as the frequency increases, the real and imaginary parts of *n*_eff_ show a decreasing trend, which is consistent with that of the graphene permittivity. Within the frequency range below 2.5 THz, the absorbance is below 90 %, which is due to the degraded impedance matching between the high-index AHSM and the free space. For TM wave excitation, although impedance matching occurs at the high frequencies, the relatively low absorbances of near 0.8 are observed in Figure 5(e). This can be attributed to the different effective interaction lengths of the electromagnetic field for two distinct metastructure orientations under the TE and TM wave excitations. For the absorption dip in Figure 5(f) and (g), the effective permittivity shows the resonance feature, whereas the magnetic permeability demonstrates the anti-resonance feature. They originate from the intrinsic property of the metamaterial with the ideal spatial periodicity [54]. Such resonance and anti-resonance features would interfere with the performance of broadband absorption and could be avoided by reducing the size of the honeycomb edge.

**Figure 6:**
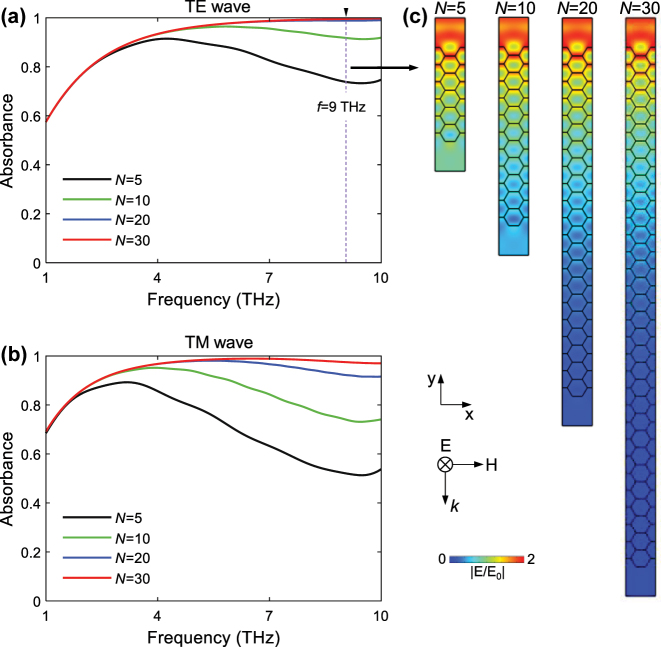
Absorbance of AHSM with different *N* values under (a) TE and (b) TM wave excitations. (c) The electric field distributions under TE wave excitation for different *N* values, where *a* = 9 μm and *μ*_*c*_ = 0.7 eV.

Based on the above discussion, we proceed to investigate the wave-absorbing performance of AHSM with a small honeycomb edge size (*a* = 9 μm), which avoids the emergence of absorption dip between 1 THz and 10 THz. Figure 6(a) and (b) indicate an overall enhancement in absorption as *N* increases from 5 to 30. For TE wave excitation, the absorbance approaches saturation when *N* exceeds 20. Conversely, achieving saturated absorption under TM wave excitation requires a larger value of *N*. We analyze the electric field distributions of AHSM at 9 THz for TE wave in Figure 6(c). The field intensity gradually decreases from top to bottom in the metamaterial. The AHSM with a larger *N* contains a greater number of graphene sheets, which enables an extended propagation path for the THz wave. This enhances the multiple reflections and destructive interferences and elevates the absorptive capacity of AHSM. Therefore, one can adopt a small honeycomb edge with the appropriate *N* number to ensure high absorption in the middle and high THz frequencies.

### 3.2 Tuning effect of graphene’s chemical potential on the absorption performance

**Figure 7:**
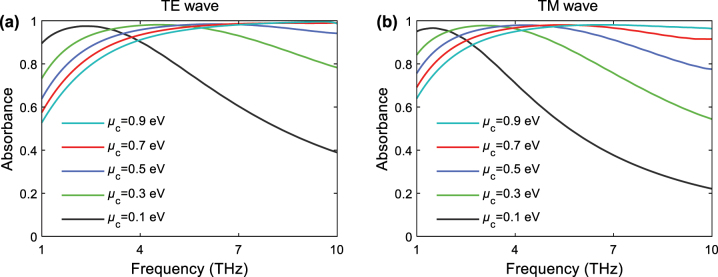
Absorbance of AHSM for various chemical potential values of graphene under (a) TE and (b) TM wave excitations, where *a* = 9 μm and *N* = 20.

Subsequently, we investigate the effects of graphene’s chemical potential *μ*_*c*_ on the absorption performance. When we fix the honeycomb edge size to 9 μm and the layer number to 20, the high absorption band gradually narrows and shifts towards the lower frequencies as *μ*_*c*_ decreases, which can be observed in Figure 7(a) and (b). These results can be attributed to two aspects according to Figure 2. On one hand, graphene mainly shows strong metallic properties in low THz frequencies. Reducing its chemical potential in this THz range can degrade its metallic features, and boost the impedance match between the AHSM and air, which facilitates the entry and absorption of THz wave within the metastructure. On the other hand, in the high THz frequency range, the imaginary part of graphene permittivity drops dramatically as the chemical potential is reduced, which weakens the THz wave absorption capability significantly. These results denote that the chemical potential of graphene provides another degree of freedom to tune the THz absorbance of AHSM, especially for enhancing the light–matter interaction in the lower THz frequencies.

### 3.3 Design of ultrawideband THz absorber

**Figure 8:**
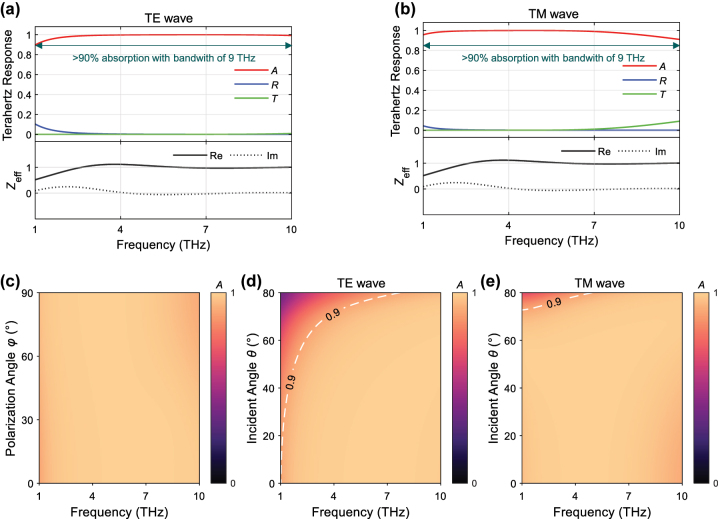
THz responses of AHSM under (a) TE and (b) TM wave excitations. (c) Absorbance spectral mapping for various polarization angles. Absorbance spectral mappings for different incident angles under (d) TE and (e) TM wave excitations, where *a* = 9 μm, *N* = 200, and *μ*_*c*_ = 0.1 eV.

The above study shows that the AHSM with a smaller honeycomb size and a larger stack layer number is more favorable for high absorption in the middle and high THz frequencies, respectively; while using a lower chemical potential of graphene is beneficial for improving THz absorption in the frequencies. Based on these principles, we design an AHSM with the parameters of *a* = 9 μm, *N* = 200, and *μ*_*c*_ = 0.1 eV, which demonstrates the high absorbance over 90 % in the ultrabroadband THz range from 1 THz to 10 THz for both TE and TM wave excitations, as depicted in Figure 8(a) and (b). According to the EMT analysis, the real part of the AHSM’s effective impedance remains close to 1, while its imaginary part approaches 0. This indicates the nearly perfect impedance matching between the AHSM and air over the entire frequency range. We further investigate the broadband absorption dependence on the polarization angle and incident angle of THz waves. The absorbance spectral mapping for various polarization angles ranging from 0° (TE wave) to 90° (TM wave) is plotted in Figure 8(c). The high absorption with the absorbance over 90 % can be observed with varying polarization angles. There are always graphene sheet layers parallel to the electric field direction of THz waves, and the AHSM absorption has good polarization insensitivity. Figure 8(d) shows the absorbance spectral mapping for various incident angles under TE wave excitation. The AHSM has a large absorption bandwidth when the incident angle changes from 0° to 60°. At the incident angle of 60°, the absorbance is above 90 % in the range from 2.3 THz to 10 THz, corresponding to a bandwidth of 7.7 THz. Under TM wave excitation, the large bandwidth with high absorbance is kept for oblique incidence, as shown in Figure 8(e). The results in Figure 8(d) and (e) exhibit that the broadband high THz absorption of AHSM has good stability on incident angle, which is attributed to the multiple reflections within the metastructure and the high Ohmic dissipation in the stacked graphene layers. Therefore, the optimal AHSM can accomplish the ultrabroadband high THz absorption for various polarizations and incident angles, which is promising in many THz applications.

Finally, to illustrate the novelty of the proposed absorber, we compare its performance with other reported THz broadband graphene-based absorbers in Table 1. Notably, the AHSM exhibits perfect absorption performance regarding absorption bandwidth. It shows the inherent advantages of graphene aerogels, such as lighter weight, lower density, higher efficiency, and simpler fabrication process. The results imply that our design has the potential to develop high-performance absorbers.

**Table 1:** Comparison of the proposed absorber with reported broadband THz absorbers.

Refs.	Structure	Bandwidth (*A* > 90 %)	Center frequency	Relative bandwidth^a^
[19]	Hybrid graphene-VO_2_ metamaterial	0.94 THz	0.805 THz	116.8 %
[21]	Graphene-Dirac semimetal metamaterial	4.2 THz	6.89 THz	61.0 %
[25]	Double-layer graphene metamaterial	3.06 THz	5.34 THz	57.1 %
[30]	3D graphene aerogel	3.5 THz	2.25 THz	155.6 %
Our work	Anisotropic honeycomb stack metamaterial	9 THz	5.5 THz	163.6 %

^a^Relative bandwidth is defined as the ratio of the bandwidth to the center frequency.

## 4 Conclusions

In summary, we propose a THz absorber using an anisotropic honeycomb stack metamaterial of graphene. Our study investigates the physics of wave absorption at different THz frequencies by employing the effective medium and impedance matching theory. To achieve high absorption at low frequencies, we propose reducing the chemical potential of graphene to degrade the metallic characteristics of the metamaterial. At intermediate frequencies, one can reduce honeycomb edge size to avoid the constructive interference of absorption dips. Furthermore, increasing the stack layer number can enhance the multiple reflection and destructive interference for elevating absorption at high frequencies. Guided by these principles, we design an ultrabroadband THz absorber with the absorbance exceeding 90 % from 1 THz to 10 THz. Notably, the absorption exhibits good tolerance to a wide range of polarization and incident angles. Our findings provide a theoretical guide to experimental studies of graphene aerogels in the future, and will also inspire more applications in the fields of THz metamaterial absorbers.
